# Exclusion of older adults and immunocompromised individuals in influenza, pneumococcal and COVID-19 vaccine trials before and after the COVID-19 pandemic

**DOI:** 10.1007/s40520-023-02380-4

**Published:** 2023-04-07

**Authors:** Katrine Bukan, Toby Pearce-Slade, Mads Eiberg, Marco Tinelli, Dafna Yahav, Jose Tuells, Olivier Epaulard, Jon G. Holler, Casper Roed, Christian Søborg, Jens-Ulrik Stæhr Jensen, Zitta Barrella Harboe

**Affiliations:** 1 Department of Pulmonary Medicine and Infectious Diseases, Copenhagen University Hospital - North Zealand, Hillerød, Denmark; 2 Department of Clinical Medicine, Faculty of Health and Medical Sciences, University of Copenhagen, Copenhagen, Denmark; 3 European Society of Clinical Microbiology and Infectious Diseases (ESCMID) Study Group for Infections in the Elderly (ESGIE), Basel, Switzerland; 4 Istituto Auxologico Italiano, IRCCS, San Luca Hospital, Milan, Italy; 5 Infectious Diseases Unit, Sheba Medical Center, Ramat-Gan, Israel; 6 European Society of Clinical Microbiology and Infectious Diseases (ESCMID) Vaccine Study Group (EVASG), Basel, Switzerland; 7 Department of Community Nursing, Preventive Medicine and Public Health and History of Science, University of Alicante, Alicante, Spain; 8 Infectious Disease Unit, Centre Hospitalier Universitaire Grenoble-Alpes, Grenoble, France; 9 Department of Respiratory Medicine, Copenhagen University Hospital, Herlev and Gentofte Hospital, Gentofte, Denmark; 10 Institute for Clinical Medicine, Faculty of Health Sciences, University of Copenhagen, Copenhagen, Denmark

**Keywords:** Vaccine trials, Exclusion, Older adults, Immunocompromised, Pneumococcal vaccine, Influenza vaccine, COVID-19 vaccine

## Abstract

**Background:**

Older adults and immunocompromised individulas are often excluded from vaccine trials.

**Aim:**

We hypothesised that during the coronavirus disease 2019 (COVID-19) pandemic, the proportion of trials excluding these patients decreased.

**Methods:**

Using the US Food and Drug Administration and and European Medicines Agency search engines, we identified all vaccines approved against pneumococcal disease, influenza (quadrivalent vaccines), and COVID-19 from 2011 to 2021. Study protocols were screened for direct and indirect age exclusion criteria and exclusion of immunocompromised individuals. In addition, we reviewed the studies with no explicit exclusion criteria and investigated the actual inclusion of those individuals.

**Results:**

We identified 2024 trial records; 1702 were excluded (e.g., use of other vaccine or risk group); and 322 studies were eligible for our review. Among the pneumococcal and influenza vaccine trials (*n* = 193), 81 (42%) had an explicit direct age exclusion, and 150 (78%) had an indirect age-related exclusion. In total, 163 trials (84%) trials were likely to exclude older adults. Among the COVID-19 vaccine trials (*n* = 129), 33 (26%) had direct age exclusion and 82 (64%) had indirect age exclusion; in total, 85 (66%) trials were likely to exclude older adults. Therefore was a 18% decrease in the proportion of trials with age-related exclusion between 2011 and 2021 (only influenza and pneumococcal vaccine trials) and 2020–2021 (only COVID-19 vaccine trials) (*p* = 0.014). In a sub-analysis assessing observational and randomised trials, the decrease was 25% and 9%, respectively. Immunocompromised individuals were included in 87 (45%) of the pneumococcal and influenza vaccine trials compared with 54 (42%) of the COVID-19 vaccine trials (p = 0.058).

**Conclusions:**

During the COVID-19 pandemic, we found a decrease in the exclusion of older adults from vaccine trials but no significant change in the inclusion of immunocompromised individulas.

## Introduction

The suddenness and duration of the coronavirus disease 2019 (COVID-19) pandemic made it impossible to ignore the disproportionately high risk of severe outcomes to older adults and immunocompromised individuals from a vaccine-preventable disease [1]. However, although these individuals are among the most susceptible to infections from many vaccine-preventable diseases, in addition to severe acute respiratory syndrome coronavirus 2 (SARS-CoV-2), they are often underrepresented in vaccine trials [2, 3]. This long-standing paradox of underrepresentation of the most susceptible populations in vaccine clinical research may result in limited external validity of the studies regarding high-risk individuals and, ultimately, in a lack of evidence to support recommendations for vaccinating the populations we are most interested in protecting.

The study populations included in clinical trials, including vaccine trials, should ideally reflect disease demographics. However, the significant heterogeneity among high-risk individuals may pose a challenge to participant recruitment and retention in clinical trials and can affect the internal validity of the trials results [4]. These factors raise legitimate concerns regarding the feasibility of including such populations in clinical research. To address the issue of disparities in the inclusion of subgroups in clinical trials [1], funding and regulatory agencies have adopted different approaches, including requirements in grant applications and progress reports to assess outcomes in specified subpopulations, especially older individuals [5–7]. Nevertheless, disparities in the inclusion of these groups remain an ongoing challenge that needs to be addressed.

A recent study [2] found that among COVID-19 treatment and vaccine trials conducted early in the pandemic (from October 2019 to June 2020), older adults were likely to be excluded from more than 50% of treatment trials and 100% of vaccine trials. We hypothesised that awareness of the increased high risk of mortality in these populations during the COVID-19 pandemic has led to a shift in addressing disparities in vaccine clinical trials, favouring the inclusion of individuals at greater risk of severe outcomes. To explore this hypothesis, we reviewed all registered clinical trials for vaccines against the three leading vaccine-preventable respiratory infections in adults (influenza, pneumococcal disease, and COVID-19) approved during the past decade and examined inclusion and exclusion criteria that directly or indirectly resulted in the exclusion of older adults and immunocompromised individuals.

## Methods

**Fig. 1:**
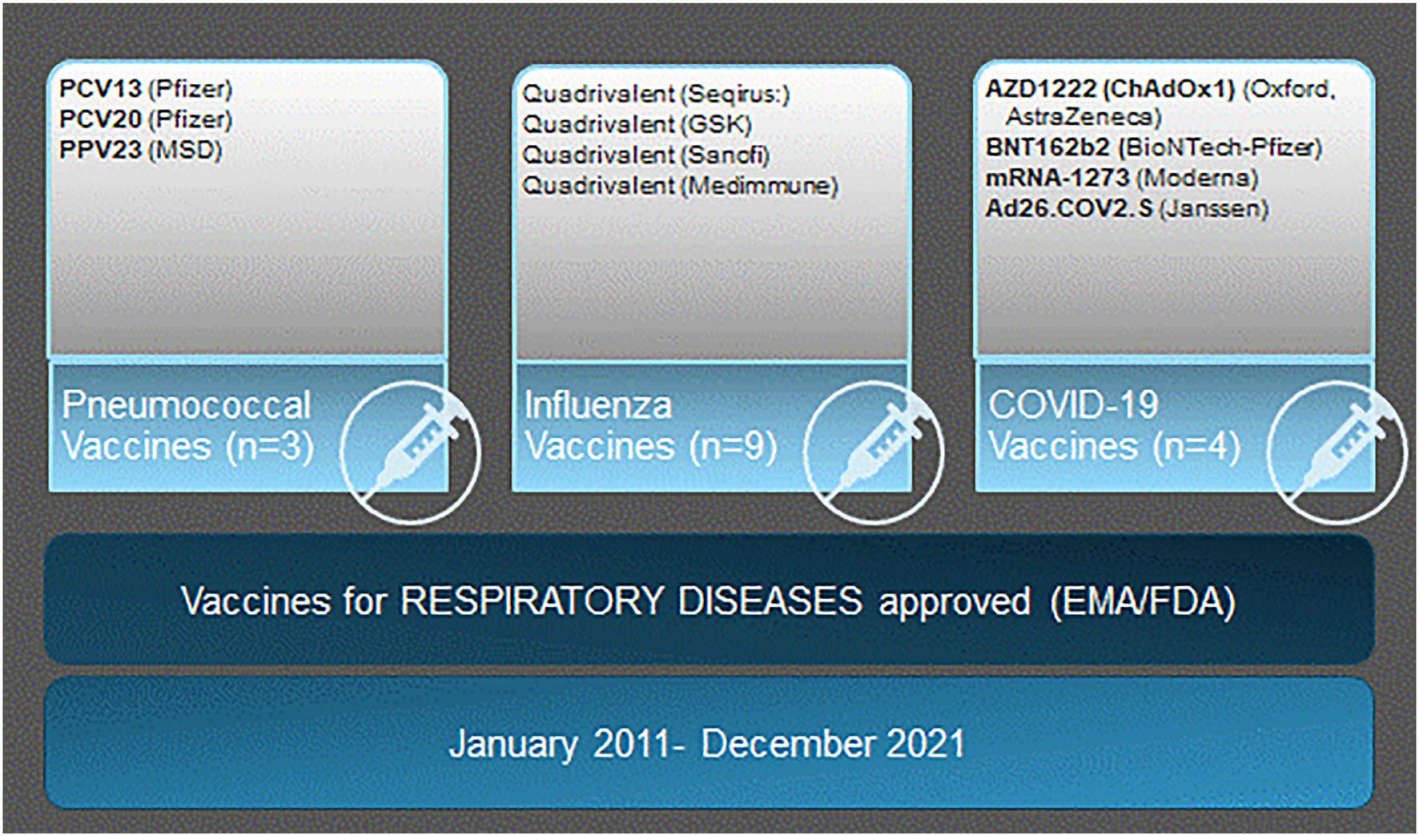
Vaccines against respiratory diseases approved for use in adults included in the systematic search. European Medicines Agency (EMA); U.S. Food and Drug Administration (FDA); 13-valent pneumococcal conjugate vaccine (PCV13); 20-valent pneumococcal conjugate vaccine (PCV20); 23-valent pneumococcal polysaccharide vaccine (PPV23). For COVID-19 vaccines, research names are indicated

**Fig. 2:**
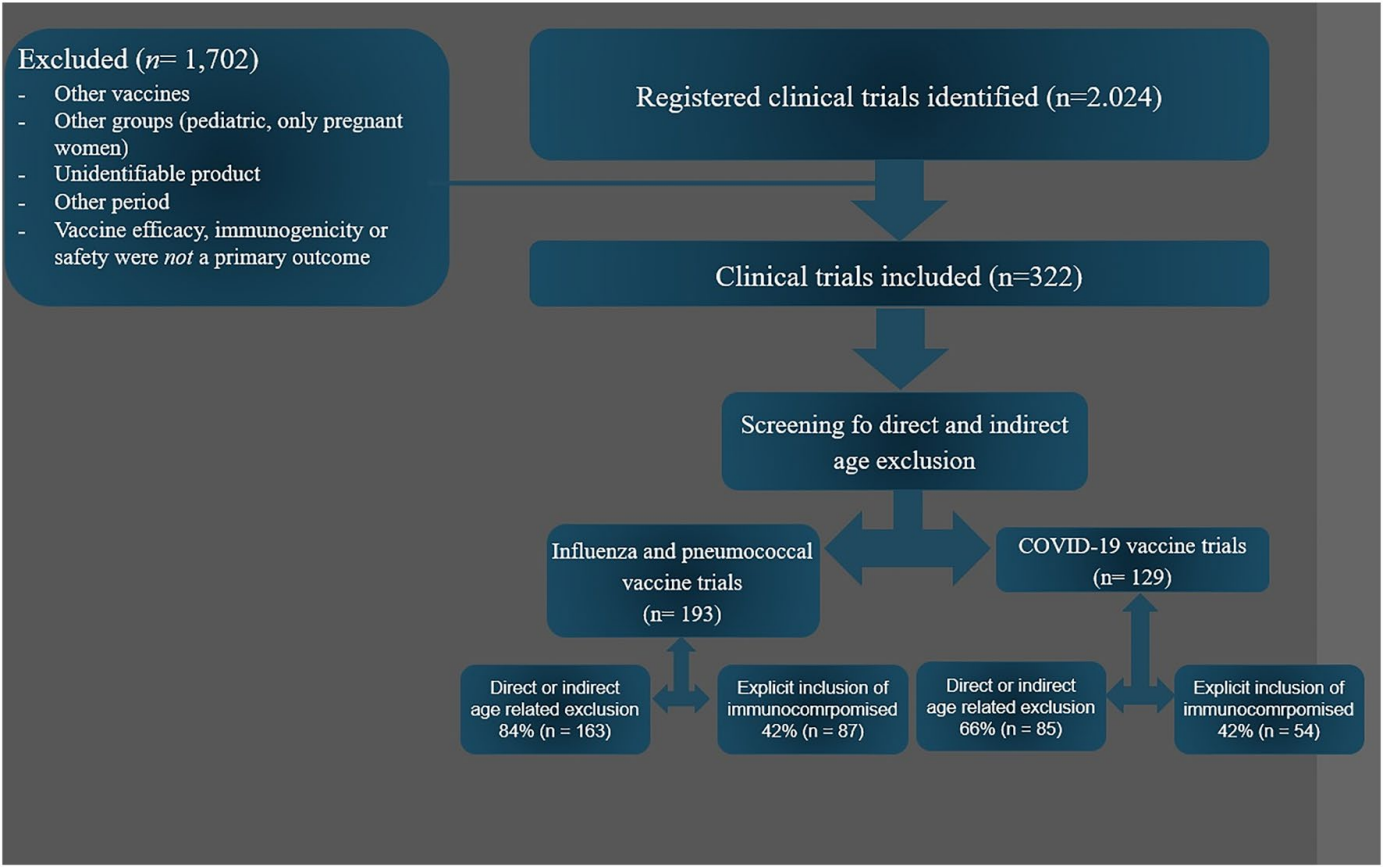
Overview of the seach and main study results

We used the European Medicines Agency (EMA) and the U.S. Food and Drug Administration (FDA) search engines [8, 9], as previously described by Helfand et al. [2]. First, we identified all vaccines against pneumococcal disease, seasonal influenza, and COVID-19 approved for adults from 1 January, 2011 to 31 December 31 2021. For influenza, we limited the review to trials investigating only quadrivalent seasonal influenza vaccines (Fig. 1). Then, we reviewed all planned, ongoing, and finalised clinical trials registered on http://www.clinicaltrials.gov, with trial enrolment initiated during the period stated above. We excluded: (1) studies in which vaccine efficacy, immunogenicity, or safety were *not* a primary outcome, (2) trials using unidentifiable vaccines, based on the information published on http://www.clinicaltrials.gov, and (3) studies that only included pregnant or lactating women or only included children (Fig. 2).

Each clinical trial record registered on the Clinical Trials’ database was reviewed by two independent investigators, who collected the information in a standardised form. A third reviewer resolved any discrepancies.

Clinical trial records were screened for direct and indirect age exclusions and for exclusions of other susceptible individuals, as previously described by Helfand et al. [2]. Studies with direct age exclusion had an explicit upper age limit for recruiting participants (e.g., none; ≥ 55–64 years; ≥ 65–84 years; ≥ 85 years). Studies with indirect age exclusion were those in which the listed exclusion criteria would disproportionately affect the inclusion of older adults. These indirect exclusion criteria included: (1) non-severe comorbidities (e.g., stable chronic conditions), (2) physical impairment ( e.g., low-performance status, frailty, residing in a long-term care facility, non-specific hearing or vision impairment), (3) cognitive impairment (e.g., dementia), (4) compliance concerns (e.g., subjects who the investigator considered unable to comply with the requirements of the protocol, or subjects with a history of alcohol or drug abuse), (5) requirements for access to or ability to manage digital platforms and technology (e.g., wireless internet, smartphone, or having access to a webcam), or 6) broad, poorly specified exclusions (e.g., vague statements such as “not in good health,” “any other criteria which, in the investigator's opinion, would compromise the subject's wellbeing or the outcome of the study” or studies enrolling only health care professionals).

Additionally, we noted the clinical trial records that explicitly excluded immunocompromised individuals, which included immunodeficient patients (e.g., those with asplenism, acquired immune deficiency syndrome, or immunoglobulin deficiencies), patients with haematological diseases or solid cancers, solid organ or stem-cell transplant recipients, as well as patients with other conditions requiring immunosuppression (e.g., rheumatic and autoimmune diseases, chronic kidney disease with or without the need for dialysis, inflammatory bowel disease, or multiple sclerosis).

We extracted the following variables: Clinical Trials database identification number, geographic area (e.g., the United States of America, Europe, or other location), trial type (multicentre or single center, randomised or non-randomised), trial phase, sample size (actual or planned), vaccine name, and date of enrolment.

All clinical trial records were categorised according to the year enrolment was initiated. For studies with multiple treatment arms, we categorized the trial according to the vaccine used in the experimental arm. For pneumococcal and influenza vaccine trials, we categorised trials into 3- or 4- -year intervals (2011–2014, 2015–2018, 2019–2021), considering 2018 as the year of implementation of the National Institutes of Health policy of inclusion across the lifespan [5]. COVID-19 vaccine trials were reported separately, in accordance with their initiation during 2020 or 2021.

For studies with no direct or indirect age-related exclusion criteria or criteria that explicitly excluded immunocompromised individuals, we reviewed the published trial results after the study was completed and recorded whether the investigators succeeded in including these groups. Trial registration records with a status corresponding to “unknown,” “withdrawn,” “not yet recruiting,” or “still recruiting” in the clinical trial registration site were excluded from this part of the analysis.

We compared proportions using the two-sample t-test; a *p-value* < *0.05* was considered statistically significant.

## Results

### Direct and indirect age-related exclusion in trials

We identified 2,024 vaccine clinical trial records, and 322 of these studies were considered eligible for our analysis (Fig. 2). The most frequent reason for excluding an influenza vaccine trials was that the trial did not use a quadrivalent influenza vaccine (*n* = 442, 22%). Among the 322 trials included in this review, 206 (64%) were randomised clinical trials, 106 (33%) were observational studies, 98 (30%) were influenza vaccine trials, 95 (30%) were pneumococcal vaccine trials, and 129 (40%) were COVID-19 vaccine trials. In total, 52% (*n* = 166) of the trials were multicentre studies, mainly initiated in the USA or Europe (*n* = 228, 71%); notably, 174 (54%) of the trials had received sponsorship from a pharmaceutical company.

**Fig. 3:**
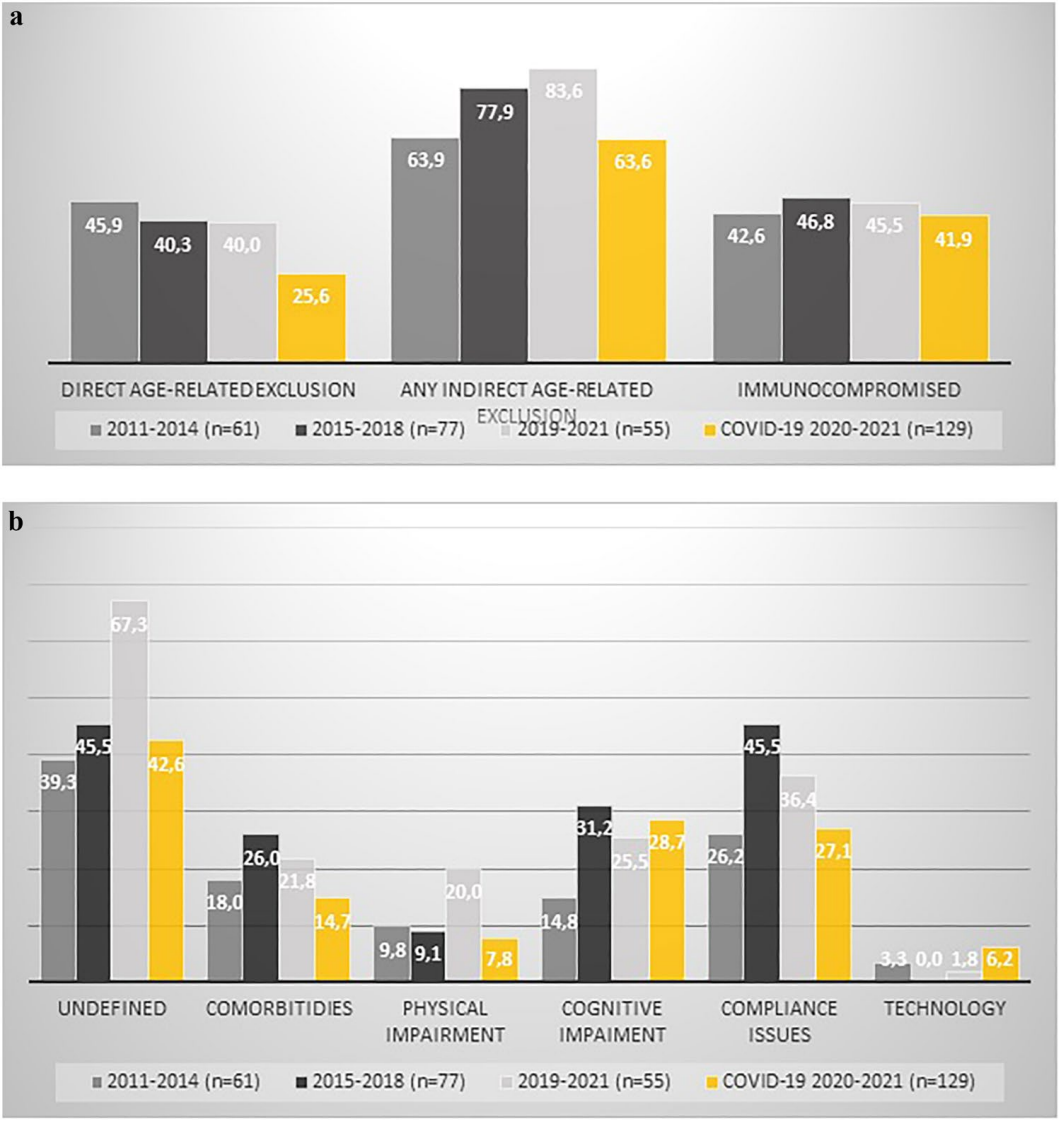
Direct and indirect age-related exclusion in included trials

Among the pneumococcal disease and influenza trials (*n* = 193, 60%), 81 (42%) had direct age-related exclusion criteria, and 150 (78%) had indirect age-related exclusion criteria (Fig. 3A). The most common indirect age-related exclusion criterion was broad, non-specified concerns (*n* = 94, 49%), followed by concerns about compliance (*n* = 71, 37%), cognitive impairment (*n* = 47, 24%), comorbidities (*n* = 43, 22%), and access to technology or digital platforms (*n* = 3, 1.5%). When direct and indirect age-exclusion criteria were combined, 163 (84%) of these trials were likely to exclude older adults.

Among the COVID-19 vaccine trials (*n* = 129, 40%), 33 (26%) had direct age-related exclusion criteria and 82 (64%) had indirect age-related exclusion critera. The most common indirect age-related exclusion criterion was broad, non-speficed concerns (*n* = 55, 43%), followed by cognitive impairments (*n* = 37, 29%), concerns about compliance (*n* = 35, 27%), comorbidities (*n* = 19, 15%), physical impairments (*n* = 10, 8%) and access to technology or digital platforms (*n* = 8, 6%) (Fig. 3B). When the direct and indirect age-exclusion criteria were combined, 85 (66%) of these trials were likely to exclude older adults.

There was an 18% decrease in the proportion of trials with direct or indirect age-related exclusion criterion between 2011 and 2021 (only influenza and pneumococcal vaccine trials) and 2020–2021 (only COVID-19 vaccine trials), and this decrease was statistically significant (*p* = 0.014). In a sub-analysis assessing the observational and randomized clinical trials separately, the decrease was 25% and 9%, respectively.

### Exclusion of immunocompromised individuals

Among the pneumococcal and influenza vaccine trials, 87 out of 193 trials (45%) explicitly included immunocompromised individuals. Among the COVID-19 vaccine trials, 54 out of 129 trials (42%) aimed to include explicitly immunocompromised individuals (Fig. 3A).

### Number of susceptible individuals included in the published trials

Among the 322 clinical trial records, there were no statements regarding the explicit exclusion of older adults in 74 studies (23%). Among these 74 studies, 55 (74%) did not explicitly report subgroup outcomes in participants older than 65 years. Only eight of the studies (10%) reported data for participants older than 65 years or for subgroups including older adult participants; six studies did not include participants older than 65 years. In addition, three studies were excluded from this analysis because the trial was withdrawn, of unknown status, or terminated (one study each).

There were 94 trial registration records in which immunosuppression was not an exclusion criterion. Among these 94 studies, 51 (54%), reported no outcome data for immunocompromised individuals. For 33 trials (35%) some or all participants were immunocompromised, usually because the study was designed to investigate this specific population. Nine of the 94 trials (10%) did not include immunocompromised individuals. At the time of our review, the clinical trial website reported that only one study had unknown status; therefore, we excluded this study from our analysis.

## Discussion

Our findings indicate vaccine trials have become less restrictive regarding the inclusion of older adults, with a significant 18% reduction in the proportion of recent vaccine trials with direct or indirect age-related exclusion criteria (i.e., the COVID-19 vaccine trials) compared with trials from the previous decade (i.e., the influenza and pneumococcal vaccine trials). However, we observed no significant changes in the inclusion of immunocompromised individuals. Unfortunately, despite more than 20 years of recommendations from researchers, regulatory agencies, and international societies [5–7] to suggest that those most at risk of severe illness should be included in clinical research, there is still an considerable evidence gap regarding the vaccination of these individuals [10, 11].

Chronological age alone is a suboptimal predictor of the risk of adverse clinical outcomes, including immunological responses to vaccination and infection [12]. In this context, indirect age-related criteria are essential and may be used as indicators of frailty and underlying comorbidities. In our review, we observed that many individuals were excluded for reasons that were unclear or not well-justified. Non-specific exclusion criteria are intended to protect more fragile adults, but otherwise eligible participants should not be excluded to make trials more convenient for investigators [2].

Our review of published trials with no explicit age-based exclusion criteria revealed that only a small proportion (10%) of these reported data from older adults. Furthermore, among those studies from which older adults were not explicitly excluded, few reported on outcomes for this particular group. The underrepresentation of older adults in vaccine trials and the difficulties associated with investigating age-specific outcomes in this group is a challenge in interpreting clinical trials’ results and implementing them in clinical practice [2]. Possible solutions to address this underrepresentation may include identifying barriers to enrolment, recognising interventions that can improve the recruitment of older participants, and generating better methods for reporting their outcomes. In addition, alternative approaches, such as providing incentives for investigators who include underrepresented populations in their trials, and requiring age-stratified data in progress reports could be beneficial [5].

Similarly, the proportion of individuals with immunocompromising conditions included in trials was low, and in approximately one-third of these trials, the study was designed to investigate a subgroup of immunocompromised individuals. Immunocompromised individuals tend to be excluded from vaccine trials for some of the reasons mentioned above. However, pharmaceutical companies should be encouraged to conduct post-licensing studies in immunocompromised individuals. If a vaccine proves effective in the general population included in the approval randomized controlled trial, a further trial to compare the vaccine against a placebo in immunocompromised individuals might not be ethical. However, studies investigating optimal vaccine dosing and scheduling in immunocompromised individuals would have merit. Alternatively, good observational studies to assess vaccine effectiveness in this group of patients could also be beneficial.

The results of our study of COVID-19 vaccine trials are encouraging. However, they underline that we must address health disparities and include key underrepresented key populations in vaccine clinical trials. Regulatory agencies played an important role in including older adults in COVID-19 vaccine trials [13]. Eliminating upper age limits for study inclusion, reducing the use of eligibility criteria that disproportionately affect multimorbid older patients, and ensuring that vaccine clinical trials are practicable for older adults will facilitate the inclusion, participation, and retention of older adults in vaccine clinical trials. Furthermore, these changes will improve access to evidence-based treatments for older adults and at-risk populations. Therefore, awareness and involvement from donors and policymakers are crucial.

One limitation of our study is that we did not conduct a detailed review of every trial protocol in detail: only trials with no explicit exclusion criteria. Furthermore, we investigated whether vaccine trials ad become more inclusive after the COVID-19 pandemic, but the period after 2020 is short. Therefore, significant changes may still appear in the coming years and we cannot be sure, that the trend towards increased inclusion of older adults will continue in future studies, as the focus on the COVID-19 pandemic decreases.

We hope that training, awareness, and commitment will increase the enrollment of older adults and other susceptible individuals to ensure that clinical vaccine trials are directly relevant to those most in need of protection.
